# How Sexuality Education Programs Have Been Evaluated in Low- and Lower-Middle-Income Countries? A Systematic Review

**DOI:** 10.3390/ijerph17218183

**Published:** 2020-11-05

**Authors:** Olena Ivanova, Masna Rai, Kristien Michielsen, Sónia Dias

**Affiliations:** 1 Division of Infectious Diseases and Tropical Medicine, Medical Centre of the University of Munich (LMU), 80802 Munich, Germany; 2 German Center for Infection Research (DZIF), Partner Site Munich, 80802 Munich, Germany; 3 Institute of Epidemiology, Helmholtz Zentrum München-German Research Centre for Environmental Health, 85764 Neuherberg, Germany; masna.rai@helmholtz-muenchen.de; 4 International Centre for Reproductive Health (ICRH), Department of Public Health and Primary Care, Faculty of Medicine and Health Sciences, Ghent University, 9000 Ghent, Belgium; kristien.michielsen@ugent.be; 5 NOVA National School of Public Health, Public Health Research Centre, Universidade NOVA de Lisboa & Comprehensive Health Research Center (CHRC), 1600-560 Lisbon, Portugal; smfdias@yahoo.com

**Keywords:** sexuality education, evaluation, systematic review, complex intervention, sexual and reproductive health, adolescent

## Abstract

Background: Complex sexual and reproductive health interventions, such as sexuality education (SE), contain multiple components and activities, which often requires a comprehensive evaluation design and adaptation to a specific context. In this review, we synthetize available scientific literature on types of evaluation designs used for SE programs in low- and lower-middle-income countries. Methods: Two databases yielded 455 publications, from which 20 articles met the inclusion criteria. Narrative synthesis was used to summarize the findings. Evaluation approaches were compared to recommended evaluation frameworks. The quality of articles was assessed by using MMAT 2018. Results: A total of 15 interventions employed in 10 countries were evaluated in the 20 selected articles, with the quality of publications being moderate to high. Randomized controlled trial was the predominant study design, followed by quasi-experimental design. There were seven process evaluation studies, using mixed methods. Main outcomes reported were of public health or behavioral nature—condom use, sexual debut or delay, and number of sexual partners. By comparing evaluation designs to recommended frameworks, few studies fulfilled at least half of the criteria. Conclusions: Evaluations of SE are largely dominated by quantitative (quasi-)experimental designs and use of public health outcomes. To improve understanding of SE program effectiveness, it is important to assess the quality of the program development, its implementation, and its impact, using existing evaluation frameworks and recommendations.

## 1. Introduction

This paper studies the designs used to evaluate sexuality education interventions in low- and lower-middle-income countries (LMICs).

### 1.1. Sexuality Education

The first International Technical Guidance on Sexuality Education published by The United Nations Educational, Scientific and Cultural Organization (UNESCO) in 2009 defined it as an “age-appropriate, culturally relevant approach to teaching about sexuality and relationships by providing scientifically accurate, realistic, non-judgmental information” [1]. Sexuality education programs aim to enhance several mutually reinforcing components: to increase knowledge and understanding; to explain and clarify feelings, values, and attitudes; to develop or strengthen skills; and to promote and sustain risk-reducing behaviors.

Sexuality education (SE) is one of the prominent examples of complex interventions, which are widely implemented in the field of sexual and reproductive health (SRH). They are frequently described as interventions that contain several interacting components. However, there are other features that make them complex, such as the number of groups and organizational levels targeted by the intervention, the degree of flexibility, and the difficulty of behaviors required by those delivering or receiving the intervention [2]. Complex interventions challenge traditional approaches regarding their design, implementation, and evaluation in different contexts [3].

In the last decade there have been multiple SE programs implemented across different settings in LMICs that illustrate the complexity of such interventions. Consider the example of the SE program implemented by Kemigisha et al. 2019 for very young adolescents, delivered by university students in primary schools, addressed multiple topics, aimed at changing SRH knowledge, well-being, and behaviors of participants, was guided by the community advisory board and had to overcome a shaky political context around sexuality education [4].

Crystalizing the causal link between multiple topics, activities, and context introduced during SE programs and changes in the young peoples’ well-being and behaviors (e.g., contraception use or decision-making skills), requires a multifaceted approach to evaluation. 

### 1.2. Recommended Evaluation Methods for Complex Interventions

The most often used and valued type of evaluation design is randomized controlled trials (RCT), which are on top of the hierarchy of evidence [5]. They are powerful to causally link an SE intervention to a certain outcome; however, they are not able to provide an understanding of the many facets of effectiveness, e.g., which component worked and why, how the intervention was conceptualized, or how it was accepted by the participants [6]. Increasingly, evaluation scientists are favoring more innovative and complimentary designs, such as process evaluation or mixed-methods evaluations, to unpack key characteristics of effective programs and highlight the multiple contextual factors and mechanisms that influence adolescent sexual behavior and well-being [7]. For instance, process (implementation) evaluation carried out in connection with a trial could help to explore how the intervention was implemented, why it succeeded, and how it can be improved [2]. This combination was suggested by Bonell et al., in 2012, as a realist RCT of complex public health interventions, which helps to examine the effects of the intervention components, to analyse pathways of change, to explore how the intervention effects vary with context, and to employ qualitative and quantitative data [8]. Process evaluations are especially relevant in multi-center trials, where the standardized intervention may be delivered, adapted, and received in different ways [9].

Some authors also suggested specific frameworks to evaluate SE interventions in terms of design, quality, implementation, and outcomes. For instance, the review and consensus on evaluation of SE programs in European countries by the European Expert Group on Sexuality Education suggested that quality and implementation of SE programs should be assessed alongside public health outcomes, such as decrease of teenage pregnancies or sexually transmitted infections (STIs) [10]. Additionally, there are a number of tools to assess content and delivery of SE programs, such as Sexuality Education Review and Assessment Tool (SERAT), Inside and Out: Comprehensive Sexuality Education (CSE) Assessment Tool or a school-level index of CSE implementation quality, by Keogh et al., 2019 [11,12,13]. 

### 1.3. Study Aim

Despite the availability of multiple evaluation frameworks and methods suitable for complex interventions, as well as suggestions on assessment of quality and implementation of SE programs, little is known on its use and applicability in different settings. The aim of this review is to synthetize available scientific literature on evaluation designs used for SE programs and to assess the actual evidence-base for SE in LMICs.

The review answers three research questions:

What are the most common evaluation designs used for sexuality education interventions?

How do these evaluations align with existing recommendations for the evaluation of complex interventions (European Expert Group on Sexuality Education and Realist Evaluation)?

What are the self-reported benefits and limitations of different evaluation designs?

## 2. Materials and Methods 

We adhered to the Preferred Reporting Items for Systematic Reviews and Meta-Analyses (PRISMA) guidelines for systematic reviews [14]. This review was registered in the PROSPERO database—CRD42020148735.

### 2.1. Search Strategy

We searched two main databases: PubMed and Web of Science. Search terms relevant to sexuality education, age groups and evaluation approaches were used. The study population of interest were adolescents and youth (10–24 years old). The UN define adolescents as individuals being 10–19 years old and youth as those persons between the ages of 15 and 24 years [15]. Only studies, which were conducted in LMICs according to The World Bank classification were included [16]. Search terms are described in Table 1. Data search was performed between April and August 2019. In addition, we completed a manual search of the reference lists of relevant articles. All records were exported into Mendeley—an online reference management program produced by Elsevier. After we removed the duplicates, titles and abstracts were screened for inclusion.

### 2.2. Study Selection

**Figure 1:**
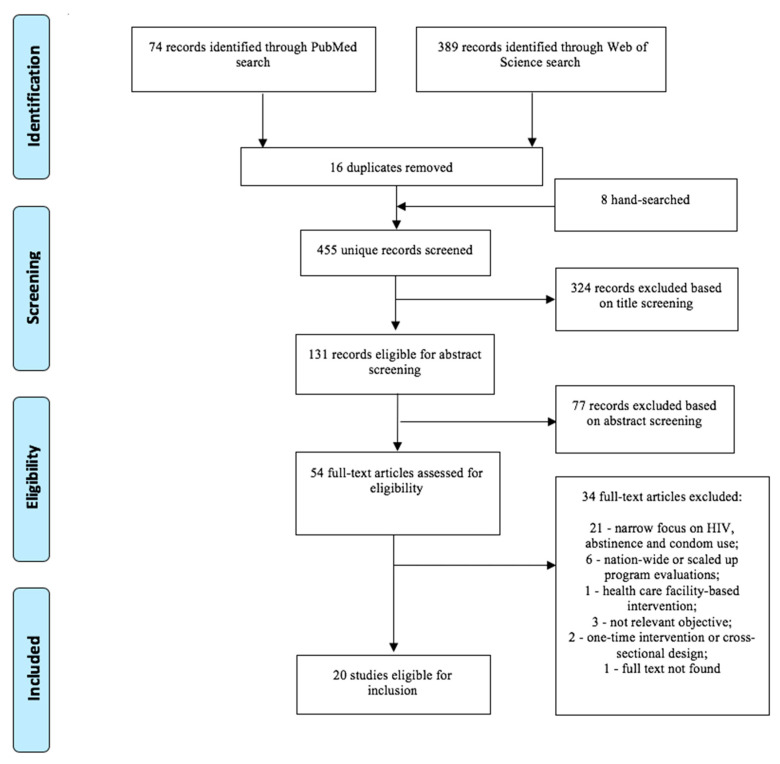
Preferred Reporting Items for Systematic Reviews and Meta-Analyses (PRISMA) flow.

This review was limited to full-text original peer-reviewed articles published in English, between January 2009, the year when UNESCO’s International Technical Guidance on Sexuality Education was published [1], and January 2019. Articles were excluded if they: (1) provided insufficient information, for example letters, abstracts or conference papers; (2) had a narrow focus on HIV-related knowledge and outcomes; (3) focused exclusively on abstinence approach to sexuality education without addressing broader topics such as contraception or other STIs; (4) evaluated only national or widely scaled-up programs, which may require more complex approach to evaluation influenced by a number of factors such as region, type of schools etc., and render its incomparable with small-scale interventions; and (5) implemented interventions exclusively in health care facilities without school or community components. Details of the study selection are summarized in Figure 1. Titles of the 455 studies and abstracts of 131 records were screened. Full texts of articles that passed the title/abstract stage were obtained for text screening.

### 2.3. Data Extraction 

We extracted data relevant to the review questions. Two authors independently read all included articles and extracted data in a predefined and pretested data extraction form in Excel. The following was extracted from each article: authors, year, study setting, main study objectives, study population, study design, limitations, and study findings.

### 2.4. Data Analysis

A descriptive narrative synthesis was chosen as the most relevant and suitable method of data synthesis for this review [17]. Additionally, we developed a framework to assess the comprehensiveness of the evaluation designs, based on realist evaluation components and recommendations for evaluating SE by the European Expert Group [10,18]. The following aspects were assessed:

- Use of a theory of change (ToC), log frame or middle-range theory (MRT);
- Use of mixed methods and data triangulation;
- Inclusion of key concepts of realist framework: context, mechanism and outcome (CMO);
- Program evaluation: age appropriateness; gender sensitivity; culturally and socially responsiveness; human rights-based approach; positive attitude towards sexuality; comprehensive content; involvement of children and youth in needs assessment and program development; quality and variety of educators’ and students’ manuals;
- Implementation evaluation: process of program development; teacher/educator training and support; linkages with relevant sexual and reproductive health services; and curriculum delivery (e.g., discrepancies in implementation);
- Outcome and impact evaluation: short-term outcomes (e.g., knowledge, reflection on norms and values etc.); evaluation by children and youth (e.g., curriculum appreciation); long-term outcomes (e.g., public health outcomes, including unintended pregnancies, and positive sexual self-perception).

Further details on definitions and description of these components are provided elsewhere [10,18]. To calculate and report overall scores for each criterion, we employed a conservative approach; we only assigned score (1), if the criterion was fully addressed and described in the article. 

### 2.5. Critical Appraisal

The quality of the included studies was assessed by using the updated mixed-methods appraisal tool (MMAT) [19]. The tool helps to examine the appropriateness of the study aim, adequacy and methodology, study design, data collection, study selection, data analysis, presentation of findings, discussions, and conclusions. For each of the included studies, the relevant five quality questions were asked corresponding to the study type, e.g., qualitative, quantitative (randomized or non-randomized trial) or mixed methods. For instance, the questions addressed were as follows: Is randomization appropriately performed? Is there an adequate rationale for using a mixed methods design to address the research question? Are the findings adequately derived from the data? and other questions depending on the study design. The studies were scored by using percentages (0–100%), where 100% is the highest score. It helped to create an overview of the quality of studies, and there was no exclusion of articles based on the quality score. Any discrepancies were discussed until a consensus was reached between two authors.

## 3. Results

From the 455 identified records, 20 studies met the inclusion criteria [20,21,22,23,24,25,26,27,28,29,30,31,32,33,34,35,36,37,38,39].

### 3.1. Critical Appraisal of Included Studies 

All publications scored 60% and more; among them, nine studies received 60%, seven studies received 80% and four studies received 100% (see Table 2). 

### 3.2. General Description of Included Interventions

Study details, methodology and the main objectives of the evaluations are presented in Table 2. Included studies were conducted in 10 countries in Africa and South America. Three interventions were multi-centered, including at least two countries. Two publications reported on quantitative evaluation of the same intervention at different time points [32,33], and seven other publications evaluated three interventions applying different evaluation designs [21,24,25,28,30,34,37]. Thus, the 20 articles included an assessment of 15 SE interventions. All evaluation studies were published between 2009–2019, however almost half of the interventions (*n* = 7) were implemented before 2009. 

All interventions were delivered primarily in schools with three having an additional community component. The sample size of participants varied, from 42 to 12,462 adolescents. The majority of interventions (*n* = 13) targeted adolescents 10–19 years old, and two also included youths of 20–24 years old. Adolescents benefited from sexuality education were of both sexes; however, three studies targeted only girls [29,35,39]. One program provided sexuality education to students with learning disabilities [27] and one to orphan adolescent girls [35].

Duration of SE programs varied. It was delivered via sessions, lectures or modules, which lasted from 35 min to 1.5 h, and were usually delivered on a weekly basis. The number of sessions and weeks differed between studies, from six to 25 sessions and from five to 16 weeks. Sexuality education was taught by teachers, educators, peers, or volunteers (local or foreign). Lectures, discussions, workshops, home assignments, plays, drama, sport events, comics, and storytelling were used to teach SRH topics. The most frequently addressed topic was HIV/STIs, followed by contraception use, delay of sexual activity, decision-making and negotiation skills, pregnancy prevention, parental communication, prevention of gender-based and sexual violence, and gender norms.

### 3.3. Evaluation Designs

Almost half of the interventions (*n* = 7) used an RCT design, with pre- and post-implementation quantitative assessment comparing an intervention and a control group. Other interventions followed a quasi-experimental design, with or without a control group, using mixed-methods, quantitative, or qualitative approaches to data collection. The majority of publications reported outcome and effectiveness evaluation results, with less focus on implementation (process) evaluations (see Table 2). Seven publications reported findings from implementation evaluations incorporated in outcome assessment (*n* = 1) or as a stand-alone assessment (*n* = 6). Nine evaluations exclusively used questionnaires (self-administered, face-to-face interviews or Audio Computer Assisted Self Interviews (ACASI)) for data collection, while the rest of the studies used a combination of different tools—questionnaires, in-depth interviews (IDIs), focus-group discussions (FGDs), biological samples, observations, checklists, cost tracking, attendance lists, and feedback forms. Evaluations targeted primarily adolescents who participated in the SE programs; however, a number of assessments (*n* = 6) also included teachers/educators, parents/caregivers, social workers, and peer educators. Evaluation outcomes were mostly reported per arm—intervention vs. control, as the predominant design was an RCT. A handful of studies disaggregated outcomes per gender.

### 3.4. Comparison of Included Evaluations Using Realist Evaluation and Expert Group Consensus Criteria

We applied a number of criteria outlined in the methodology section, to assess how the included studies made use of and incorporated them into their evaluation designs (see Table 3). While several publications reported on behavioral theories, Intervention Mapping, community engagement, and evidence used to develop study activities, only a handful of studies (*n* = 4, from which one partially and three fully) developed and published a theoretical framework to demonstrate mechanisms on how their intervention activities aimed to address the expected outcomes and to illustrate the specific context. As described in the section above, half of the evaluations applied exclusively quantitative methods to assess the outcomes, while the other half applied mixed-methods approach to data collection (*n* = 6). A total of four evaluations (three partially and one fully) mentioned context and/or mechanisms and/or outcomes (CMO) to indicate how and which mechanisms were activated by implemented interventions and in what conditions, to reach the desired outcomes. 

Program and implementation criteria, e.g., age appropriateness of the program, rights-based approach, and interactive teaching, were partially addressed by all evaluations. All studies measured outcomes (short-term), e.g., improved SRH knowledge, self-esteem and skills developed, with almost half also addressing impact (long-term), such as reduction in STIs and sexual violence. However, the majority of studies demonstrated short-term outcomes immediately after implementation period and up to 24 months, and only one study looked at the longer period—54 months post-intervention [32]. Main outcomes reported were of public health or behavioral nature—condom use, sexual debut or delay, number of sexual partners, STIs incidence, number of unintended pregnancies, and service or HIV/STIs testing usage. Some studies looked at the improvement in SRH knowledge and attitudes, while others looked at communication on SRH-related topics with parents or peers. Seven process (implementation) evaluations reported on design of the intervention, dose, fidelity, acceptance of the intervention, barriers and facilitators of implementation, and monitoring and evaluation processes.

### 3.5. Self-Reported Limitations and Benefits of Different Evaluation Designs

Publications addressed mostly limitations of the study designs. As RCT with a quantitative assessment was used in almost half of the interventions, the main limitations inherent to it were as follows:

- Loss to follow-up and low response rate;
- Recall and self-reporting bias;
- Contamination and systematic differences between intervention and control groups;
- Length of intervention—short with no long-term follow-up;
- Underestimation of the intervention effect due to provision of benefits to control group;
- Low statistical power to perform sub-analysis, e.g., gender or dose, and challenges to pair pre- and post-measurements due to missing data or intervention adherence issues;
- Questionnaire-related issues, e.g., language, terminology and scales used;
- Lack of data triangulation.

Generalizability of findings was also questioned by many authors and non-randomized design was seen as a limitation per se. In case of multicomponent interventions, e.g., Aninanya et al.2015, it was impossible to determine—by using pre- and post-intervention survey—which component or components most influenced study outcomes [31]. Studies that used mixed-method or qualitative approaches reported researchers’ bias and lack of representation from different groups, e.g., interviews only with educators and not students.

A handful of studies reported benefits of different evaluation designs and tools used. The strong points were mostly related to RCT design, such as randomization, retention and use of face-to-face interviews/ACASI; however, it was clear from the discussions that mixed-method approach, involvement of various stakeholders, and contextualization of findings hold a potential of strengthening and enriching any evaluation design.

## 4. Discussion

To our knowledge, this is the first systematic review to summarize available peer-reviewed evidence on evaluation designs used for complex SE interventions in LMICs. This review not only describes evaluation designs used with their limitations and benefits, but it also compares them to the recommended evaluation frameworks for complex interventions, such as realist evaluation and consensus on evaluation of SE programs. 

Randomized control trial (RCT) and quasi-experimental designs with pre- and post-measurements were predominately applied to interventions reported in this review. Similar reviews also demonstrated that these designs are still considered as a “gold standard” for outcome and effectiveness evaluations [10,40]. However, the authors included in the review mentioned multiple limitations related to these designs, such as randomization and blinding, short-term follow-up, drop-out rates, and low external validity [6].

Another shortcoming highlighted is the need for a large sample size to demonstrate a desired effect, which is costly and requires a multi-region or national program implementation [41]. Further, one more potential pitfall of using RCT is the desire to fit the intervention into the “gold standard” and recommended evaluation design, instead of the other way around. Such approach may compromise the quality of the intervention, hinder context adaptation in multi-center trials and prevent from depicting other relevant outcomes, besides of biological or public health outcomes. Similar concerns were also raised by the European Expert Group on Sexuality Education [10]. 

Additionally, while experimental designs can provide estimates of SRH intervention effectiveness, they offer limited insights on how and why the intervention worked or not. Having only an outcome evaluation result does not allow to distinguish how different components or content were adapted and delivered in practice. They also provide little insight into the ways through which interventions lead to behavior change and what were the facilitators and barriers in these processes. As a result, the ability to generalize and compare findings from one study to a different context might be compromised. Studying the impact mechanisms by using, for example, program and process evaluations alongside trial designs, provides valuable additions and a better understanding of planning, implementation, and monitoring of SRH interventions. The lack of such studies is demonstrated by findings from the current review, where only seven articles used process evaluation or reported on feasibility and acceptability of the intervention. Moreover, using qualitative methods alongside quantitative approach offers more insights into behavioral change in young people receiving sexuality education intervention.

This review also demonstrated that research of SE effectiveness is mostly focused on the reduction of risky behaviors, e.g., STI or unwanted pregnancies as public health outcomes. Secondary outcomes are mostly describing a change in SRH knowledge and attitudes. There is a very limited use of indicators that focus on positive aspects of sexuality. Despite the fact, that indicators such as self-efficacy are often used, they are usually only considered in respect to the desired behavior change, and not as a stand-alone. Indicators measuring the ability to experience pleasurable and satisfying sexual relationships are seldomly used [10]. The updated UNESCO International Technical Guidance on Sexuality Education also highlighted limited rigorous studies assessing “non-health” outcomes to date [42]. 

A review by Lopez et al. 2016 found that trials do not always adequately report the content of interventions [40], and Hoffmann et al., in 2014, suggested that the overall quality of description of interventions in publications is notably poor [43]. We also faced this challenge when conducting our review, as a handful of studies reported, in detail, the topics addressed and activities performed. This hindered eligibility for a number of studies. There is a need to have a detailed description of the intervention, especially if the evaluation tries to identify a component which has contributed the most to the success of the intervention. 

Until around 2009, sexuality education was mainly focused on the issues of HIV infection, risk reduction, and abstinence. A slight shift in terminology, content and perspective on SE took place after UNESCO technical guidelines in 2009 [44]. However, half of the evaluated interventions in this review were implemented before the guidelines became available; thus, the definition and components of sexuality education varied among the studies. We excluded the studies with a narrow focus on HIV and abstinence-only aspect; however, it was challenging to judge from the intervention descriptions to what extent other topics, e.g., decision-making skills and gender or rights, were equally integrated in the curriculum and delivered. To improve the reporting standards, tools such as the Template for Intervention Description and Replication (TIDieR) could be used [43]. In addition, a handful of studies reported on development and use of theory of change (ToC) or log frame, which helps to illustrate the activities and links to desirable outcomes and impact. This is an essential step for any outcome and impact evaluation, which guides the implementation process and assists in design of the evaluation [45].

Few studies in this review conducted SE interventions in multiple contexts. Leveraging heterogeneity through testing an intervention in different settings and performing in-depth case studies might strengthen applicability of the findings [46]. At the same time, the heterogeneity of SE content, delivery, implementation, and evaluation is seen between world regions and countries. The majority of peer-reviewed evidence on SE is coming from high-income countries (HICs). Thus, this review targeted sexuality education programs in LMICs, where adolescents’ SRH indicators, social, cultural, and political contexts differ from that in HICs, such as the USA and the European Union member states. For example, in 2016, an estimated 68% of adolescent girls aged 15–19 in LMICs have completed seven or more years of education, with higher rates in Latin America and lower in Africa (51%) [47]. Thus, non-governmental organizations (NGOs) and out-of-school settings in these countries might play a stronger role in implementation of SE. Simultaneously, conservative opposition to SE, lack of teacher training, political will, financing, strong monitoring, and evaluation mechanisms exist in many LMICs and HICs [48,49,50].

To summarize, based on the results of this review, we can demonstrate that SE programs are describing short-term outcomes (*n* = 14) well; however, we cannot make strong conclusions on whether the SE programs and their curricula were of a good quality, nor whether they were implemented in a high-quality manner. Finally, we have little insights into how the included SE programs meant to achieve their outcomes, as very few (*n* = 3) provided ToC, log frame, or MRT.

### Limitations

This systematic review has a number of limitations. Firstly, only studies published in English were considered, leading to the exclusion of studies published in other languages, such as Spanish, French, or Russian, which are widely spoken in many low- and lower-middle-income countries around the globe. Secondly, this review did not include grey literature, such as UN reports and studies conducted by NGOs, which do not often make it into the peer-reviewed literature and, potentially, use approaches other than RCT approaches. Thirdly, the MMAT appraisal tool was used to assess the quality of reporting in the studies, but more specialized quality assessment tools, such as the Cochrane Collaboration’s tool for assessing risk of bias, could have provided more in-depth reviews of quality. Additionally, specific search terms yielded a moderate number of articles, thus studies where “sexuality education” or “evaluation” were not specifically mentioned in a title/abstract or substituted by broad terms, such as “school-based intervention”, “SRH program”, “HIV intervention”, “design and implementation”, etc., might be missed. Lastly, due to time constraints and workload, we performed search in two databases: PubMed and Web of Science, which are the most often used search databases; however, we might have missed some relevant studies included in other databases, e.g., Global Health or EMBASE. Finally, we used a conservative approach to calculate overall scores in Table 3–only fully (Y) met criteria. Thus, such approach could misclassify some interventions, as it was not always clear from the information provided in the articles to what extent each criterion was addressed.

## 5. Conclusions

This review demonstrated a lack of mixed-methods, theory-driven, and comprehensive approaches in the evaluation of complex sexuality education program. While randomized control trials and quasi-experimental designs are undoubtedly important to demonstrate intervention effectiveness, they are not sufficient to comprehensively evaluate complex interventions. There should be a space for flexibility and adaptability of the evaluation designs to the intervention theory, content, and context. The need for the quality assessment of the development, implementation, and effectiveness of the sexuality education in different settings remains.

## Figures and Tables

**Table 1:** Search terms used.

Characteristic	Search Terms Combined with AND
Study population (adolescents and youth)	(adolescent OR adolescents OR adolescence OR girl OR boy OR youth OR teenage OR teen OR young woman OR young man OR young boys OR young girl OR young women OR young men OR young person OR young people OR student OR pupil OR learner OR young female OR young male OR young adult)
Evaluation	(evaluation OR assessment OR impact evaluation OR outcome evaluation OR process evaluation OR realist evaluation OR formative evaluation OR randomized trial OR qualitative evaluation OR quantitative evaluation OR effectiveness evaluation OR summative evaluation OR quasi-experimental design OR non-randomized trial OR pre-post evaluation OR before-after study evaluation OR randomized design OR non-randomized design OR qualitative design OR cost-effectiveness analysis OR economic evaluation)
Sexuality education	(sexuality education OR sex education OR abstinence education OR reproductive education OR family values education OR life skills education OR family life education OR sexual health education OR reproductive health education)
Low and lower-middle income countries	(Africa OR Asia OR Latin America OR South America OR Central America OR Central Asia OR Eastern Europe OR South Asia OR South East Asia OR Former Soviet Union OR Afghanistan OR Benin OR Burkina Faso OR Central African Republic OR Chad OR Comoros OR Congo OR Eritrea OR Ethiopia OR Gambia OR Guinea OR Guinea-Bissau OR Haiti OR Korea OR Liberia OR Madagascar OR Malawi OR Mali OR Mozambique OR Nepal OR Niger OR Rwanda OR Senegal OR Sierra Leone OR Somalia OR South Sudan OR Syrian Arab Republic OR Tajikistan OR Tanzania OR Togo OR Uganda OR Yemen OR Zimbabwe OR Angola OR Bangladesh OR Bhutan OR Bolivia OR Cabo Verde OR Cambodia OR Indonesia OR Kenya OR Kiribati OR Kosovo OR Kyrgyz Republic OR Lao PDR OR Papua New Guinea OR Philippines OR São Tomé and Principe OR Solomon Islands OR Sri Lanka OR Sudan OR Cameroon OR Côte d’Ivoire OR Djibouti OR Egypt OR El Salvador OR Georgia OR Ghana OR Honduras OR India OR Lesotho OR Mauritania OR Micronesia OR Moldova OR Mongolia OR Morocco OR Myanmar OR Nicaragua OR Nigeria OR Pakistan OR Swaziland OR Timor-Leste OR Tunisia OR Ukraine OR Uzbekistan OR Vanuatu OR Vietnam OR West Bank and Gaza OR Zambia)

**Table 2:** Description of studies and evaluation designs.

N	Author	Year of Publication	Country	Study Design	Setting	Target Population of the Intervention	Evaluation Design	Target Population of the Evaluation	Objective of the Evaluation	Data Collection Tools Used for the Evaluation	MMAT %
1	Aninanya	2015	Ghana	RCT	School and community	female and male adolescents (10–19)	pre- and post-quantitative	female and male adolescents (10–19)	to measure the impact of the intervention on SRH service usage and satisfaction	questionnaire	60%
2	Dunbar	2014	Zimbabwe	RCT	Study center and community	female orphan adolescents (16–19)	pre- and post-quantitative	female orphan adolescents (16–19)	to measure increase of SRH knowledge, improvement in social and economic indicators, reduction of risky behaviors, HIV acquisition and unintended pregnancy	Audio Computer Assisted Self Interviews (ACASI) and face to face interviews	80%
3	Gaughran	2014	Kenya	non-RCT, no control	School	female adolescents (13–21, mean age = 16.5)	pre- and post-mixed method	female adolescents (13–21, mean age-16,5)	to evaluate students’ knowledge, attitudes and self-efficacy and the efficacy of the curriculum	questionnaire, IDIs and FGDs	80%
4	Hanass-Hancock	2018	South Africa	non-RCT, pilot	School	female and male adolescents with learning disabilities	qualitative (implementation evaluation)	educators	to understand educator’s perspectives and experiences with using the curriculum in their classrooms	IDIs	100%
5	Harrison	2016	South Africa	non-RCT, pilot, control	School	female and male adolescents (14–17)	pre- and post-quantitative	female and male adolescents (14–17)	to measure changes in condom use, partner communication, gender beliefs and values; perceived peer behaviors; self-efficacy for safer sex	questionnaire (2–3 interviewers read the questions aloud in class)	60%
6	Ivanova	2016	Bolivia, Ecuador and Nicaragua	non-RCT, no control	School and community	female and male adolescents	qualitative (process evaluation)	female and male adolescents, parents, health care providers, peers, project team	to study additional outcomes of the intervention not studied by the initial evaluation; to identify problems and facilitating factors in the design, implementation, monitoring and evaluation of the intervention that may have influenced its outcomes	IDIs and FGDs	100%
7	Jemmot *	2015	South Africa	RCT	School	female and male adolescents (mean age = 12.4)	pre- and post-quantitative	female and male adolescents (mean age-12.4)	to report the intervention’s effects on sexual behaviors (sexual intercourse, condom use etc.) and STIs during a 54-month post-intervention period	questionnaire, urine and blood samples	100%
8	Jemmot *	2010	South Africa	RCT	School	female and male adolescents (mean age = 12.4)	pre- and post-quantitative	female and male adolescents (mean age-12.4)	to report the intervention’s effects on sexual behaviors (sexual intercourse, condom use etc.) and STIs during a 3,6 and 12-month post-intervention period	questionnaire	100%
9	Katahoire	2018	Uganda	RCT	School	female and male adolescents (12–15) and parents/caregivers	pre- and post-quantitative	female and male adolescents (12–15), parents/caregivers	to evaluate the effects of a school delivered sexuality communication intervention designed to increase frequency and improve quality of parent/caregiver-adolescent sexuality communication	questionnaire	60%
10	Klinger	2015	Madagascar	non-RCT, no control	School	female and male adolescents (15–19)	pre- and post-quantitative	female and male adolescents (15–19)	to evaluate the immediate impact of the curriculum on SRH knowledge, attitudes and self-efficacy	questionnaire	60%
11	Krugu	2018	Ghana	RCT	School	female and male adolescents and youth (10–21)	pre- and post-quantitative	female and male adolescents and youth (10–21)	to test the effects of an intervention on SRH knowledge, attitudes and risk perception	questionnaire	60%
12	Mathews	2016	South Africa	RCT	School	female and male adolescents (mean age = 13)	pre- and post-quantitative (incorporated process evaluation-data on fidelity, exposure and acceptability)	female and male adolescents (mean age-13)	to test the effect of the intervention to delay sexual debut, increase condom use and decrease intimate partner violence	questionnaire, observations and attendance register	60%
13	Mathews **	2012	South Africa and Tanzania	RCT	School	female and male adolescents (12–14)	pre- and post-quantitative	female and male adolescents (12–14)	to assess the effect of the intervention on delaying sexual debut and condom use	questionnaire	60%
14	Merrill	2018	South Africa	non-RCT, no control	School	female adolescents (11–16)	pre- and post-mixed-method and process evaluation	female adolescents (11–16)	to investigate changes in short-term outcomes defined in the intervention model immediately before and after intervention delivery; to understand the intervention’s implementation, including the quantity and quality of the intervention; to examine mechanisms of impact, including participants’ responses to and unintended consequences of the intervention; and to explore contextual factors that facilitate or impede intervention delivery	participant attendance, SMS platform usage tracking, questionnaire, structured observations, FGDs and IDIs.	80%
15	Mukoma **	2009	South Africa	RCT	School	female and male adolescents (12–14)	mixed method (process evaluation)	female and male adolescents (12–13), teachers	to assess whether the intervention was implemented as planned; to assess the quality of the implementation; to understand the impeding and enabling factors for implementation; to assess acceptability and subjective evaluations of the intervention among the students and teachers; and to provide information that could assist in the interpretation of the behavioral outcomes.	observations, teacher lesson logs, IDIs, FGDs	80%
16	Namisi **	2015	South Africa and Tanzania	RCT	School	female and male adolescents (12–16)	pre- and post-quantitative	female and male adolescents (12–16)	to examine to what extent a school-based HIV prevention education program led to higher levels of interpersonal communication between adolescents and adults about sexuality issues	questionnaire	60%
17	Rijsdijk ***	2011	Uganda	non-RCT, control	School	female and male adolescents (mean age = 16)	pre- and post-quantitative	female and male adolescents (mean age-16)	to assess the effects of intervention on the main socio-cognitive determinants (knowledge, beliefs, attitudes, perceived social norms, self-efficacy, risk perception and intention) of safe sex behavior (delaying sexual intercourse; condom use and non-coercive sex)	questionnaire	80%
18	Rijsdijk ***	2014	Uganda	non-RCT, control	School	female and male adolescents (mean age = 16)	mixed method (process evaluation)	teachers	to examine factors associated with dose delivered (number of lessons implemented) and fidelity of implementation (implementation according to the manual), as well as to identify the main barriers and facilitators of implementation	questionnaire and IDIs	80%
19	van der Geugten ****	2015	Ghana	non-RCT, no control	School	female and male adolescents and youth (12–23)	pre- and post-quantitative	female and male adolescents and youth (12–23)	to obtain more insight into the knowledge, attitudes and behavioral intentions of students concerning SRH, and to study the effects of an SRH program on this group	questionnaire	60%
20	van der Geugten ****	2014	Ghana	non-RCT, no control	School	female and male adolescents and youth (12–27, mean 17.8)	mixed method (process evaluation)	female and male adolescents and youth (12–27), educators	to examine students’ opinions on an SRH program and to explore the facilitators and barriers for educators regarding the implementation of the program	questionnaires and IDIs	80%

*Legend:* *, **, ***, **** evaluations of the same intervention; FGDs, focus group discussions; IDIs, interviews (structured, in-depth or unstructured); SRH, sexual and reproductive health; RCT, randomized control trial.

**Table 3:** Comparison to recommended frameworks for complex interventions.

N	Evaluation Study(s) per Intervention	ToC/Log Frame/MRT	Mixed Methods (Data Triangulation)	CMO	Program Quality Criteria	Implementation Quality Criteria	Outcome Criteria	Impact Criteria	Overall Score per Intervention *
1	Aninanya 2015	N	N	N	P	P	N	Y	1/7
2	Dunbar 2014	Y	N	N	P	P	Y	Y	3/7
3	Gaughran 2014	N	Y	N	P	P	Y	N	2/7
4	Hanass-Hancock 2018	NA	N	P	NA	P	Y	N	1/7
5	Harrison 2016	N	N	N	P	P	Y	Y	2/7
6	Ivanova 2016	Y	Y	P	P	P	Y	Y	4/7
7	Jemmot 2010 Jemmot 2015	N	N	N	P	P	Y	Y	2/7
8	Katahoire 2018	N	N	N	P	P	Y	N	1/7
9	Klinger 2015	N	N	N	P	P	Y	N	1/7
10	Krugu 2018	P	N	N	P	P	Y	N	1/7
11	Mathews 2016	N	N	N	P	P	Y	Y	2/7
12	Mukoma 2009 Mathews 2012 Namisi 2015	N	Y	P	P	P	Y	Y	3/7
13	Merrill 2018	Y	Y	Y	P	Y	Y	N	5/7
14	Rijsdijk 2011 Rijsdijk 2014	N	Y	N	P	P	Y	N	2/7
15	Van der Geugten 2014 Van der Geugten 2015	N	Y	N	P	P	Y	N	2/7
Overall score per criteria *		3/15	6/15	1/15	0/15	1/15	14/15	7/15	

*Legend:* N—No; Y—Yes, fully; P—partially or only few; ToC—theory of change; MRT—middle-range theory; CMO—Context–Mechanism–Outcome; NA—not applicable or not available; * overall scores based only on number of *Y—Yes, fully.*

## Abbreviations

ACASI	Audio Computer Assisted Self Interviews
FGD	focus group discussion
HIV	human immunodeficiency virus
IDI	in-depth interview
MMAT	mixed-methods appraisal tool
NGO	non-governmental organization
PRISMA	Preferred Reporting Items for Systematic Reviews and Meta-Analyses
RCT	randomized controlled trial
SE	sexuality education
SRH	sexual and reproductive health
STI	sexually transmitted infection
UNESCO	the United Nations Educational, Scientific and Cultural Organization
